# A recommendation for the use of electrical biosensing technology in neonatology

**DOI:** 10.1038/s41390-024-03369-z

**Published:** 2024-07-08

**Authors:** Lizelle van Wyk, Topun Austin, Bernard Barzilay, Maria Carmen Bravo, Morten Breindahl, Christoph Czernik, Eugene Dempsey, Willem-Pieter de Boode, Willem de Vries, Beate Horsberg Eriksen, Jean-Claude Fauchére, Elisabeth M. W. Kooi, Philip T. Levy, Patrick J. McNamara, Subhabrata Mitra, Eirik Nestaas, Heike Rabe, Yacov Rabi, Sheryle R. Rogerson, Marilena Savoia, Frederico Schena, Arvind Sehgal, Christoph E. Schwarz, Ulrich Thome, David van Laere, Gabriela C. Zaharie, Samir Gupta, ESPR Special Interest Group on Non-Invasive Cardiac Output Monitoring, Topun Austin, Bernard Barzilay, Maria Carmen Bravo, Morten Breindahl, Christoph Czernik, Eugene Dempsey, Beate Horsberg Eriksen, Jean-Claude Fauchére, Elisabeth M. W. Kooi, Philip T. Levy, Patrick J. McNamara, Subhabrata Mitra, Eirik Nestaas, Heike Rabe, Yacov Rabi, Sheryle R. Rogerson, Marilena Savoia, Frederico Schena, Arvind Sehgal, Christoph E. Schwarz, Ulrich Thome, Gabriela C. Zaharie, Samir Gupta, Lizelle van Wyk, Willem-Pieter de Boode, Willem de Vries, David van Laere

**Affiliations:** 1 Department of Paediatrics and Child Health, Faculty of Medicine and Health Sciences, Stellenbosch University and Tygerberg Hospital, Cape Town, South Africa; 2 Neonatal Intensive Care Unit, Rosie Hospital, Cambridge University Hospitals NHS Foundation Trust, Cambridge Biomedical Campus, Cambridge, UK; 3 Neonatal Intensive Care Unit, Assaf Harofeh Medical Center, Tzrifin, Israel; 4 Department of Neonatology, La Paz University Hospital and IdiPaz, Madrid, Spain; 5 Department of Neonatology, Copenhagen University Hospital, Rigshospitalet, Copenhagen Denmark; 6 Department of Neonatology, Charité - Universitätsmedizin Berlin, Berlin, Germany; 7 Department of Paediatrics and Child Health, University College Cork, Cork, Ireland; 8 Department of Neonatology, Radboud University Medical Center, Radboud Institute for Health Sciences, Amalia Children’s Hospital, Nijmegen, The Netherlands; 9 Division of Woman and Baby, Department of Neonatology, University Medical Centre Utrecht, Wilhelmina Children’s Hospital, Utrecht University, Utrecht, The Netherlands; 10 Department of Paediatrics, Møre and Romsdal Hospital Trust, Ålesund, Norway; 11 Clinical Research Unit, Norwegian University of Science and Technology, Trondheim, Norway; 12 Department of Neonatology, University Hospital Zurich, University of Zurich, Zurich, Switzerland; 13 Division of Neonatology, Department of Pediatrics, Beatrix Children’s Hospital, University of Groningen, University Medical Centre, Groningen, The Netherlands; 14 Department of Newborn Medicine, Boston Children’s Hospital, Boston, MA USA; 15 Department of Pediatrics, Harvard Medical School, Boston, MA USA; 16 Department of Paediatrics, University of Iowa, Iowa City, IA USA; 17 Institute for Women’s Health, University College London, London, UK; 18 Institute of Clinical Medicine, Faculty of Medicine, University of Oslo, Oslo, Norway; 19 Clinic of Paediatrics and Adolescence, Akershus University Hospital, Lørenskog, Norway; 20 Brighton and Sussex Medical School, University of Sussex, Brighton, UK; 21 University of Calgary, Alberta, Canada; 22 Newborn Research Centre, The Royal Women’s Hospital, Melbourne, VIC Australia; 23 Department of Obstetrics and Gynaecology, The University of Melbourne, Melbourne, VIC Australia; 24 Neonatal Intensive Care Unit, S Maria Della Misericordia Hospital, Udine, Italy; 25 Ospedale Maggiore Policlinico, Milano, Italy; 26 Monash Newborn, Monash Children’s Hospital, Melbourne, VIC Australia; 27 Department of Paediatrics, Monash University, Melbourne, VIC Australia; 28 Department of Neonatology, Center for Pediatric and Adolescent Medicine, University of Heidelberg, Heidelberg, Germany; 29 Division of Neonatology, Department of Pediatrics, University of Leipzig Medical Centre, Leipzig, Germany; 30 Neonatal Intensive Care Unit, Universitair Ziekenhuis, Antwerp, Belgium; 31 Neonatology Department, University of Medicine and Pharmacy, Iuliu Hatieganu, Cluj -Napoca, Romania; 32 Department of Engineering, Durham University, Durham, UK; 33 Division of Neonatology, Department of Pediatrics, Sidra Medicine, Doha, Qatar

## Abstract

**Abstract:**

Non-invasive cardiac output monitoring, via electrical biosensing technology (EBT), provides continuous, multi-parameter hemodynamic variable monitoring which may allow for timely identification of hemodynamic instability in some neonates, providing an opportunity for early intervention that may improve neonatal outcomes. EBT encompasses thoracic (TEBT) and whole body (WBEBT) methods. Despite the lack of relative accuracy of these technologies, as compared to transthoracic echocardiography, the use of these technologies in neonatology, both in the research and clinical arena, have increased dramatically over the last 30 years. The European Society of Pediatric Research Special Interest Group in Non-Invasive Cardiac Output Monitoring, a group of experienced neonatologists in the field of EBT, deemed it appropriate to provide recommendations for the use of TEBT and WBEBT in the field of neonatology. Although TEBT is not an accurate determinant of cardiac output or stroke volume, it may be useful for monitoring longitudinal changes of hemodynamic parameters. Few recommendations can be made for the use of TEBT in common neonatal clinical conditions. It is recommended not to use WBEBT to monitor cardiac output. The differences in technologies, study methodologies and data reporting should be addressed in ongoing research prior to introducing EBT into routine practice.

**Impact statement:**

- TEBT is not recommended as an accurate determinant of cardiac output (CO) (or stroke volume (SV)).
- TEBT may be useful for monitoring longitudinal changes from baseline of hemodynamic parameters on an individual patient basis.
- TEBT-derived thoracic fluid content (TFC) longitudinal changes from baseline may be useful in monitoring progress in respiratory disorders and circulatory conditions affecting intrathoracic fluid volume.
- Currently there is insufficient evidence to make any recommendations regarding the use of WBEBT for CO monitoring in neonates.
- Further research is required in all areas prior to the implementation of these monitors into routine clinical practice.

## Introduction

### Hemodynamic monitoring

Cardiac output (CO) is considered a fundamental physiological parameter for diagnosis and guidance of therapy in various neonatal conditions^1^. Maintaining optimal perfusion and oxygenation is of prime importance in the neonatal intensive care unit (NICU). Comprehensive monitoring of various physiological variables is required, as low CO has been associated with increased morbidity, adverse neurodevelopmental outcome, and increased mortality^2^.

The circulatory system of neonates is significantly different from that of adults or children, as the neonatal population is a heterogeneous mix of gestational and postconceptional ages, with different degrees of cardiovascular maturation^3^. Indirect measures of CO, such as HR and blood pressure (BP), are inadequate for a comprehensive assessment of neonatal hemodynamic status^4^. Comprehensive hemodynamic monitoring, including CO, is thus an essential part of neonatal care to prevent adverse outcomes.

### Cardiac output monitoring technology

CO measurement, via invasive techniques, e.g., intermittent pulmonary artery thermodilution and Fick’s method, are considered the gold standards for accurately determining CO in adults^5^. However, in neonates these methods are inappropriate^6^ as catheters are often too big and the invasiveness of these methods have been questioned^7^. Minimally invasive CO monitoring technologies encompass devices not requiring the insertion of a pulmonary artery catheter, e.g., pulse contour, pulse power analysis, partial gas re-breathing and transpulmonary ultrasound dilution^8^. Some of these technologies require the placement of an arterial line (pulse contour and pulse power analysis) and may need placement of a central venous line for calibration purposes^6^. These technologies have been poorly studied in the neonatal population whilst others are still under development (transpulmonary ultrasound dilution)^9^. Most other CO measurement methodologies in neonates offer only intermittent measurement values as they are labor, skill or technology intensive (transthoracic echocardiography (TTE) and cardiac magnetic resonance imaging)^6^.

Non-invasive CO monitoring technologies were therefore developed to overcome these challenges, offering fully non-invasive methods of monitoring stroke volume (SV) and CO. These included intermittent measurements via transcutaneous Doppler ultrasound (Ultrasound Cardiac Output Monitor and TTE) and continuous measurements via various electrical biosensing technologies (EBT) (bioimpedance (BI) and bioreactance (BR)).

For a new technology to be safely used in the clinical environment and to allow therapeutic decisions to be based upon it, it must be proven to be accurate and precise. A good agreement between a new and a reference technology is defined by a small bias (indicating a high accuracy), narrow limits of agreement (indicating a high precision) and a percentage error ≤30% (indicating technology interchangeability)^10,11^. Trending ability (change over time) should also be assessed to ensure that the new technology’s direction and magnitude of change is in line with that of the reference technology^12^.

### Overview of EBT technology

The first type of non-invasive cardiac monitoring, rheocardiography, was developed in 1949 by Kedrov^13^ but only found popularity in 1966 when Kubicek re-designed it for use in the aerospace industry^14^. Since then, numerous iterations of this technology have become available in the healthcare industry, with methodologies measuring changes in whole body, segmental or thoracic impedance from which SV and hence CO is derived. Numerous nomenclatures are used—whole body electrical bioimpedance, thoracic electrical bioimpedance (TEB), electrical velocimetry, electrical cardiometry, impedance cardiometry, impedance cardiography, thoracocardiography, bioreactance and rheocardiography. These have subtle differences, often with proprietary algorithms and models to estimate SV and CO.

For EBT a high frequency, low amplitude electrical current is applied across the thorax (TEBT) or entire body (WBEBT). The resistance (impedance, Z0) to this electrical current varies between different tissues in the body, with the primary distribution being to the blood and extracellular fluid. This change in electrical current (∆Z0) over time (dZ0/dt) corresponds to SV, from which CO can be estimated.

EBT is divided into 2 broad categories: (1) bioimpedance (BI) which encompasses thoracic electrical velocimetry, electrical cardiometry, impedance cardiography as well as WBEBT, and (2) bioreactance (BR).

Significant differences exist between BI and BR^15^ (Table 1).

**Table 1:** Comparison of technological differences between bioimpedance and bioreactance.

	Bioimpedance	Bioreactance
Assumptions regarding blood flow	Related to the change in body’s resistance only	Related to change in body’s resistance, capacitance, and inductance
Anatomical assumption regarding thorax	Fluid-filled cylinder or truncated cone	Thorax is an electrical circuit with resistor and capacitor
SV estimation	Calculated from estimated changes in resistance/ impedance (Z0) – electrical current flow over time due to aortic blood flow changes	Calculated from phase shift, determined from resistance and capacitance [amplitude (magnitude of impedance)]and phase direction of impedance (Z0)
SV calculation	Instantaneous rate of change in Z0 is related to aortic blood flow and SV is proportional to the maximal rate of change of Z0 (dZ0/d*t*_max_) and the ventricular ejection time (VET)	Peak rate of change of the phase shift (dφ/d*t*_max_) is proportional to the peak aortic flow from which SV is calculated (Eq. 2)
Electrode placement	Four electrodes – 2 outer and 2 inner – placement dependent on technology used Measurements stated not to be sensitive to placement	Applied at the base of the neck (thoracic inlet) and costal margins (thoracic outlet) on both sides. Measurements are sensitive to electrode placement
VET determination	VET is determined by the distance between QRS complexes	VET is determined by BR (first and second zero crossing of the dφ/d*t* signal) and electrocardiographic signals (peak of the QRS complex)

Modified from ref. ^15^.

*dZ0/dt* impedance change over time, *VET* ventricular ejection time, *Z0* impedance, *dφ/dt*_*max*_ peak rate of change od change of phase shift.

## Thoracic electrical biosensing technology

### Bioimpedance (BI)

#### Electrical velocimetry and electrical cardiometry

**Fig. 1:**
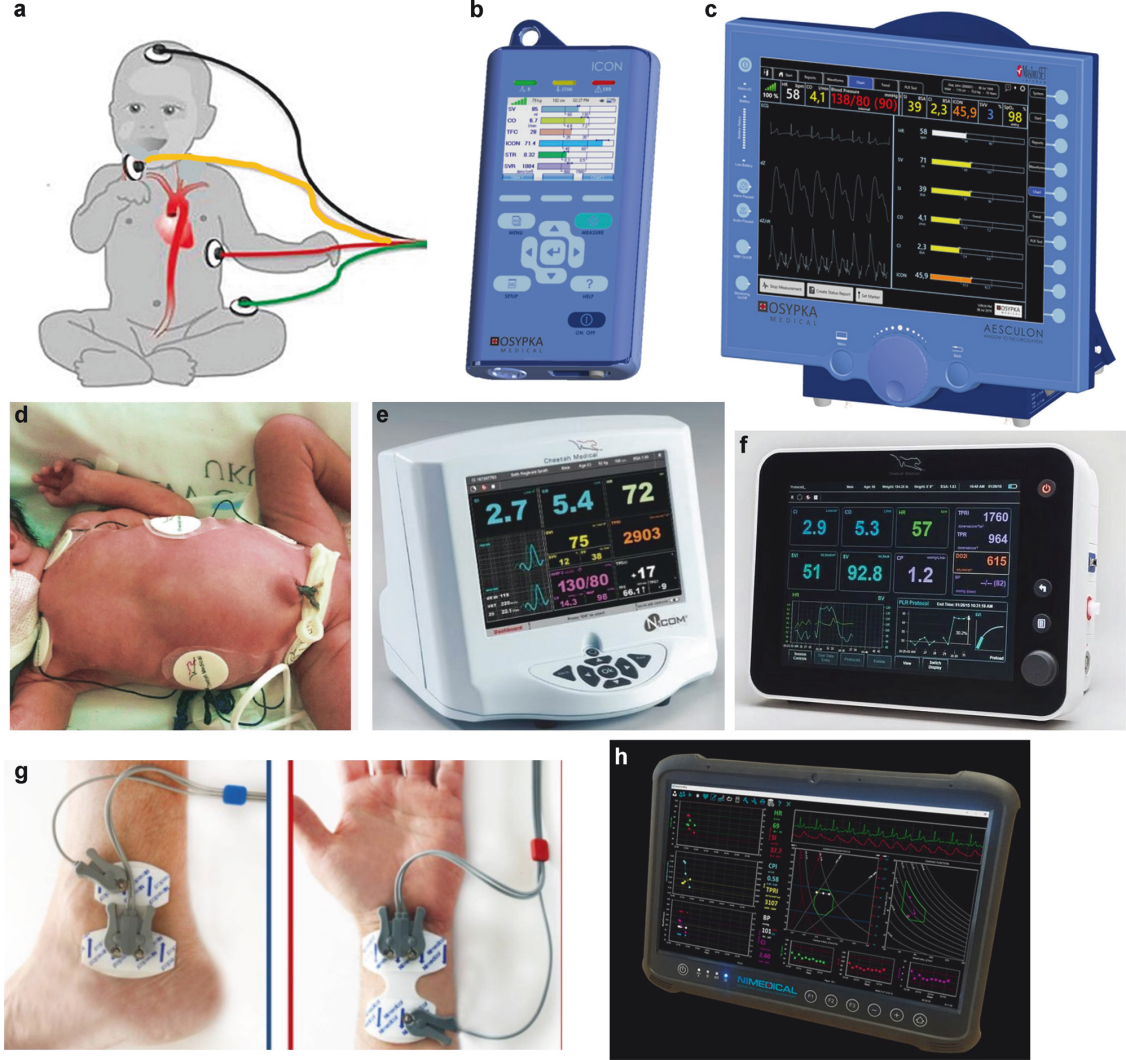
Currently available TEBT technologies. **a** Thoracic bioimpedance sensor placement; (**b**) ICON; (**c**) Aesculon; (**d**) bioreactance sensor placement; (**e**) NICOM Reliant (discontinued); (**f**) NICOM Starling; (**g**) whole body bioimpedance sensor placement; (**h**) NICaS.

Electrical cardiometry (EC) is the method of non-invasive CO technology that utilizes the model of thoracic electrical velocimetry (EV) to determine SV and CO^16^. These are used by Aesculon™ and ICON™, manufactured by Osypka Medical GmbH, Germany (Fig. 1)

In EC, the change in impedance (∆Z0) is due to the degree of erythrocyte alignment in the aorta throughout the cardiac cycle. During diastole, as the aortic blood flow ceases, erythrocytes are randomly orientated and interfere with electrical conduction. During systole as the ventricles contract, the erythrocytes are forced to align parallel to pulmonary and aortic flow and the electrical current in the large vessels passes with less impedance, hence an increased conductivity in the absence of turbulent flow. These pulsatile changes in volume and thus in impedance, in relation to the cardiac cycle (∆Z0(t)), are used to calculate ejection/ flow time and thus SV.

EV estimates SV by means of the following equation^17^:

1 $${{{\rm{SV}}}}_{{{\rm{TEB}}}}={{{\rm{C}}}}_{{{\rm{P}}}}.{{{\rm{v}}}}_{{{\rm{FT}}}}.{{\rm{FT}}}$$

where SV_TEB_ is SV estimated by TEB, C_P_ is the patient constant (volume of electrically participating tissue, in ml), v_FT_ is the mean blood velocity index (in s^-1^) during flow time (FT; measured in s). The EV model estimates SV based on the input of the patient’s body mass, an empiric means velocity index derived from a peak amplitude measurement assumed to be the peak aortic blood flow acceleration and a measurement of flow time.

*Impedance cardiography (ICG) and electrical cardiometry (EC)* are similar as both rely on periodical volumetric changes in the aorta to determine SV and CO. However, ICG and EC differ in the model applied to determine impedance measurements, specifically as to how the change in impedance is calculated. In ICG the change in impedance (conductivity) (ΔZ(t)) is solely attributed to the volumetric expansion of the ascending aorta due to the increase of volume within the aorta or due to its wall motion. The index of peak velocity of the volumetric change is used in ICG as compared to the index of peak acceleration in EV. EV includes direction of flow whereas ICG does not. In EV, volume changes also incorporate the alignment of erythrocytes^18^.

### Bioreactance (BR)

In BR, another thoracic EBT method, it is assumed that blood flow changes are not only related to changes in impedance but also changes in capacitance (ability of biological tissue to store an electrical current) and inductance (biological tissue’s ability to store energy in a non-electrical form). BR therefore measures phase shift (φ) of an oscillating current as it traverses the thorax^19^. Four pairs of sensors, one electrode acting as a high frequency generator and the other as a receiver, are placed on either side of the thorax (Fig. 1). CO measurements are determined separately from each side of the body and the final CO is the average of the measurements.

BR uses the following formula to estimate SV^20^:

2 $${{\rm{SV}}}={{\rm{C}}}\times {{\rm{VET}}}\times {{\rm{d}}}{{\rm{\varphi }}}/{{{\rm{dt}}}}_{\max }$$

where C is a constant of proportionality, VET is ventricular ejection time, and dφ/ dt_max_ is the peak rate of change of the phase shift (Δφ). BR is used by the Reliant® and its newer version, Starling®, manufactured by Baxter, Deerfield, Illinois.

## WBEBT

WBEBT is a derivative of ICG where the electrical current is passed through the whole body by the placement of electrodes on the radial aspect of the wrist and the posterior tibial area of the ankle (Fig. 1). With placement of the electrodes on the distal portions of the extremities, the low voltage current (30 K kHz AC current, 1.4 mA) passes through all major arteries and veins and the resistive portion of bioimpedance is measured.

The original NICaS monitor was upgraded to the NICaS 2004 Slim model in order to improve the accuracy and reliability of the CO, cardiac index (CI) results and their derivatives.

The various commercially available TEBT and WEBT technologies are summarized in Table 2.

**Table 2:** Commercially available EBT technologies.

Technology	Commercial name, manufacturer	Method	Sensor placement
TEBT	ICON, Osypka Medical GmbH, Berlin, Germany	BI	4 ECG electrodes Left leg, left chest, left neck, and forehead or cheek
	Aesculon, Osypka Medical GmbH, Berlin, Germany	BI	2 ECG electrodes on left chest and 2 on left side of neck
	Starling, Cheetah Medical, Baxter Medical, UK	BR	4 double electrodes
WBEBT	NICaS, NI Medical, Israel	BI	Radial artery of wrist and posterior tibial artery of ankle

*BI* bioimpedance, *BR* bioreactance, *TEBT* thoracic bioimpedance sensing technology, *WEBT* whole body biosensing technology.

### Cardiac output monitoring principles

Worldwide, 11% of all births are preterm with prematurity being the cause of 50% of all neonatal deaths^21^. With the growing number of surviving preterm neonates, at an ever-increasing younger gestational age^22^, the need for accurate monitoring is essential. The incidence of hemodynamic compromise is unknown as an exact definition is lacking. Often, blood pressure is the only parameter used and the definition of a “normal” blood pressure and the definition of hypotension is fraught with uncertainty^23,24^. Isolated, episodic clinical examination, vital signs and laboratory values are insufficient^25^ to assess a system that is in a continuous state of change, such as the neonate’s cardiovascular system^26^. For this reason, continuous, objective hemodynamic monitoring is essential.

Non-invasive CO monitoring offers the ability to continuously monitor several hemodynamic variables that provide insight into the changing dynamics of the preterm neonate’s cardiovascular system. By monitoring heart rate (HR), oscillometric blood pressure (BP) and peripheral saturation (SpO_2_) non-invasive CO monitors provide similar data to conventional vital signs monitor. In addition, these devices are able to provide SV, CO, total peripheral resistance, and thoracic fluid content (TFC), allowing estimation of global blood flow and cardio-pulmonary interaction. This may aid in determining the underlying pathophysiology of hemodynamic compromise.

The American College of Critical Care Medicine emphasizes the need for the early recognition of symptoms and the initiation of goal-orientated, time sensitive interventions to improve patient outcomes in neonatal shock. The guidelines also support the use of hemodynamic parameters, such as cardiac index (CO corrected for body surface area)^27^. In this regard, non-invasive CO monitoring may assist in the recognition of hemodynamic instability and shock, allowing the timely initiation of therapy and allowing therapeutic monitoring. The National Institute of Child Health and Human Development (NIHCD) has also emphasized the need for accurate, reliable and continuous methods to measure CO at the bedside whilst stating that TEBT-methods require validation^28^.

The use of hemodynamic monitoring in critically ill patients is reliant on the following principles: (1) no hemodynamic monitoring technique can improve outcome by itself; (2) monitoring requirements may vary over time and may depend on local availability and training; (3) there are no optimal hemodynamic values that are applicable to all patients; (4) variables should be combined and integrated; (5) CO is estimated but not measured; (6) monitoring hemodynamic changes over short periods of time is important; (7) continuous measurements of all hemodynamic variables is preferable and (8) non-invasiveness is not the only issue^29^.

Various theoretical, hardware, and patient-related factors must be considered when choosing a hemodynamic monitor (Table 3). Desirable characteristics of CO monitoring technologies are accuracy, precision, reproducibility, operator independence, rapid response time, continuous monitoring, ease of use and application, and cost effectiveness^15^. Currently, no such device exists for any patient population. The choice of CO monitor then depends on machine availability, patient characteristics, clinical situation, and practitioner preference^30^.

**Table 3:** Factors for consideration when choosing a hemodynamic monitor.

Practical considerations	Hardware considerations	Patient-related considerations
Safety & side-effects (skin due to sensors)	Accuracy/reproducibility of parameters	Influenced by cardiac rhythm, function & valvular disease
Versatility, number, relevance & utility of parameters	Rapid response time to interventions & accurate trending ability	Influenced by mechanical ventilation: tidal volume, frequency, PEEP
Able to be utilized by nurses & physicians (operator independence)	Expertise proven personal, colleagues, and literature	Type, severity & stage of disease warranting hemodynamic monitoring (e.g., shock, acute lung injury)
Ease of use and application, user-friendliness, education, learning curve	Uniformity of applicability: different patients, clinical situations, hemodynamic states	Type of circulatory support & change contemplated therein: fluids, drugs, devices
Possibility of assessing fluid responsiveness, goal-directed therapy, and other resuscitation strategies of proven outcome benefit despite no decrease in mortality	Continuous vs intermittent measurements	Vascular access & other anatomical factors (contra-indications)
Demonstrated treatment alterations	Invasive vs non-invasive	Patient tolerance
Acceptable cost-effectiveness	Availability	
	Level of integration with existing monitors	

Adapted from refs. ^15,30^.

## Clinical applications of EBT in neonatology

The Special Interest Group (SIG) on Non-invasive Cardiac Output Monitoring (NICOM) of the European Society for Paediatric Research (ESPR) deemed it necessary to provide guidance on the use of NICOM technology in the field of neonatology, based on literature up to January 2024.

Recommendations and suggestions are provided based on the available strength of evidence, as determined by the quality, quantity, and consistency of studies and evidence^31^. Recommendations were stated if there was strong evidence in the field (both positive or negative for strong or poor evidence), whereas suggestion was used based on less evidence.

Numerous studies in the neonatal setting have demonstrated the ease of use, non-invasiveness, and non-intrusiveness of the use of TEBT (supplementary data). These studies have shown changes in SV and CO in various clinical circumstances, physiological as well as pathophysiological, and during various medical and surgical interventions (supplementary data). Despite the probable lack of accuracy and precision of these devices, EBT may be able to provide additional, continuous monitoring that might be used in conjunction with traditional vital signs and monitoring aids (TTE).

## TEBT to measure cardiac output

Numerous studies have reported on the agreement and accuracy of TEBT^32–49^ (supplementary data). Various narrative and meta-analyses have shown relative accuracy (low bias), poor precision (wide limits of agreements), and a high PE, indicative of non-interchangeability with TTE of EBT in neonates^45,47,49–51^. TEBT CO has poor correlation with TTE-derived RVO^38^ as compared to TTE-LVO.

TEBT has also been shown to have poor trending accuracy over the first 72 hours^49^ and first month^45^ after birth. TEBT has been shown to longitudinally track CO, enabling the identification of low CO post PDA ligation^39^, but bias may increase over time^52^.

TEBT has been compared to TTE in most neonatal research. No studies have been performed to compare TEBT to more accurate CO measuring methods, as many are difficult in neonates (cardiac MRI) or utilize inappropriate equipment (thermodilution). The utilization of TTE as a reference method remains problematic, as it is known to be inaccurate as compared to thermodilution^1^.

Machine learning has also been used to predict the subsequent 60 min of CO with good accuracy based on the first 300 min after its application^53^.

### Recommendation

TEBT cannot accurately determine CO in neonatal care and is not interchangeable with TTE.

Its ability to longitudinally monitor CO may be clinically useful but requires further research prior to routine use.

## TEBT to determine and monitor PDA management

PDA is a common occurrence in NICU and preterm neonates. Its natural history, management, and long-term follow-up remains a challenge^54^. Pharmacological management entails indomethacin, paracetamol (acetaminophen) and ibuprofen. Surgical ligation remains a viable option for unstable neonates.

Numerous studies have included PDA as a primary or secondary outcome in EBT studies (supplementary data). The presence of a PDA has been shown to affect the accuracy of TEBT measurements, as compared to TTE^40,41,43,46,48^. Hemodynamically non-significant PDA had low bias, wide LOA and an acceptable PE^40^ whilst a hemodynamically significant duct showed a large bias, wide LOA and an unacceptable PE^40^. In contrast, the ongoing NOAH trial (Noninvasive Objective Assessment of Hemodynamics in preterm infants) showed no difference in accuracy between measurements with or without a PDA, whether hemodynamically significant or not^47^. Increased TEBT-CO within the first 24 h after birth, and low BP, has been shown to predict a PDA requiring treatment^55^.

TEBT accuracy and precision has been shown to worsen over time in preterm neonates undergoing PDA ligation, but TEBT monitoring was able to determine CO patterns for those infants who developed post ligation cardiac syndrome requiring milrinone^39^. TEBT has also been used to demonstrate a drop in CO after PDA ligation (metal clip as well as suture ligation) with recovery at 24 and 48 h post-operative, influenced by birth weight and LA/Ao ratio^56^. Various studies have shown differing drops in CO post PDA ligation (25–73%), irrespective of type of closure^56–58^.

TEBT-monitored hemodynamic variables (CI) in medical vs surgical PDA management in ELBW infants^59^ showed a better tolerance of medical closure compared to surgical closure. TEBT may be able to identify CO differences in neonates who respond to medical therapy^60^.

### Recommendation

TEBT measurements are affected by the presence of a PDA. TEBT should not be used to determine appropriate management strategies for PDA until further research has been performed.

TEBT may be helpful in monitoring longitudinal CO changes but requires further research prior to routine use.

## TEBT to monitor transition

In order to recognize low flow states, leading to hemodynamic compromise, a baseline from which to work is required. TTE-LVO proposed lower limit is 150 ml/kg/min^61^ but values vary widely^62^. Attempts have been made to determine normal values for TEBT SV and CO^42,44,63,64^. Studies have used various technologies (BI, BR, and older versions of these) with varying values determined for CO and SV. Clinicians and researchers should therefore be cognizant that there may be technology-related differences in normal values.

Transition from an intra-uterine to an extra-uterine environment remains a time of extreme changes in cardiovascular and pulmonary systems. Preterm infants remain at risk of a failure in transition due to their immature myocardium and are at risk of poor contractility in combination with the sudden decrease in systemic vascular resistance^4^. Few technologies are available to either monitor hemodynamic changes in real-time for all changes associated with transition or to provide early intervention^65^.

Various studies have utilized TEBT during the transition period (supplementary data). Studies have examined the feasibility of using TEBT in the transitional period^66–68^. Despite claiming feasibility, studies have shown the difficulty in maintaining a good signal^68,69^.

Studies monitoring CO during the first 15 min after birth have inconsistent results. Studies have shown a decrease in CO over the first 10 min after birth whilst others have shown an increase with subsequent decrease over the first few minutes^67,70,71^. Similarly, studies have shown contradictory reasons for improved CO after birth. Some have shown that SV remained stable, suggesting that the CO changes were driven by an increase in HR^67,68,70^ whilst other studies have shown that CO increase was driven by SV increases rather than HR^72,73^. Data also showed possible sex-related differences in the transitional phase, with higher CO in male infants^66,70^. The effect of resuscitation (including positive pressure ventilation or cardiopulmonary resuscitation) on TEBT-derived CO and SV has not been evaluated.

In an older TEBT-methodology study^66^, TEBT parameters did not differ between infants born via vaginal or cesarean section delivery, whilst a newer study showed differences in SV variation (SVV) between term and late preterm infants born via cesarean section and normal vaginal delivery^74^.

An older TEBT methodology study showed the ability of TEBT to monitor CO and SV in infants of diabetic mothers, but showed no difference in CO or SV, despite infants presenting with cardiomegaly^75^.

TEBT has been used to determine CO changes during delayed cord clamping (DCC) showing contrasting effects on CO^76–79^.

Desaturations and/ or bradycardia events in preterm very low birth weight infants in the first 72 h after birth have been shown to decrease CO and increase SVR, but were influenced by antenatal doppler abnormalities, gestational age, and the presence of a hemodynamically significant PDA on the changes on CO. The investigators suggested a targeted and individualized approach for minimizing cerebral injury in preterm infants^80^.

### Suggestion

TEBT may be able to monitor the transitional phase, but normal values are technology-specific and require further research. Signal quality may affect the efficacy of TEBT to monitor this period. Further studies should be performed in different gestational age brackets to determine the exact trend in CO and SV in the transitional period, and thus its application in detecting maladaptation.

## TEBT for monitoring ventilation management

Positive pressure ventilation is known to affect hemodynamics by decreasing systemic venous return, increasing right ventricular load, decreasing pulmonary blood flow, and possibly leading to systemic compromise^81^. Hemodynamic monitoring during ventilation in neonates, especially if preterm, may be beneficial.

Numerous studies have shown the effect of various modes of ventilation on the accuracy of TEBT, as compared to TTE^38,40,43,44,47,48^ (supplementary data). Evidence is contradictory regarding the degree of bias and effect on precision^48^ and the effect on the interaction between ventilation and PDA^40^. Studies have shown the largest inaccuracies were during the use of high frequency ventilation with bias increasing with increasing complexity of ventilation (nCPAP, SIMV, and HFO)^48^.

No differences in TEBT-CO or SV were found when switching between pressure controlled and volume targeted ventilation in extremely low gestational age neonates^82^. TEBT-CO has been shown to decrease post-extubation, primarily due to changes in SV due to the presence of a PDA^83^.

In a term infant study, TEBT hemodynamic indices were shown not to differ between infants with and without respiratory distress^84^. Late preterm infants who received surfactant showed a higher increase in CO and SV compared to those who had not received surfactant during the transitional phase^72^.

In an older TEBT technology study, impedance (Z0) was shown to increase with a concomitant decrease in SV and CO during the development of a pneumothorax, suggesting the ability to use TEBT to monitor for pneumothoraces^85^.

### Recommendation

TEBT cannot be used to monitor hemodynamics during invasive or non- invasive ventilation management. TEBT may be helpful in assessing longitudinal CO changes with ventilatory changes. More research is required in this area.

## TEBT to monitor sepsis and septic shock

Sepsis-related cardiovascular dysfunction is varied in neonates depending on the level and progress of sepsis. SVR may be high or low depending on the level of vasodilation leading to hypotension, as well as producing varying levels of cardiac output^86^. EBT multi-parameter monitoring may therefore be helpful in neonatal sepsis.

TEBT monitoring of HR, SVR, and CO has been stated to facilitate inotrope choice in neonatal septic shock as well as monitor response to management^87^, although TTE confirmation was not obtained to confirm changes in TEBT measurements.

TEBT monitoring of the hemodynamic status during sepsis in late preterm infants showed that SV, CO, and CI increased on day 2 after the diagnosis of sepsis as compared to controls^88^. TTE was used for confirmatory measurements, with low bias, LOA, and PE based on CI measurements.

### Recommendation

TEBT should not be used to diagnose or monitor management of septic shock in neonates. TEBT may be helpful in monitoring longitudinal CO changes during diagnosis and management of sepsis. More research is required before routine implementation.

## TEBT to monitor red blood cell (RBC) transfusions

Preterm infants with symptomatic anaemia may have a high CO and the degree of anemia has been correlated with CO^89^. RBC transfusions may therefore alter CO and monitoring would be advantageous to ensure continued adequate perfusion.

Few studies utilizing TEBT during RBC transfusions have been performed (supplementary data). TEBT studies have shown contradictory effects on CO - no CO changes^90^ as well as significantly lower CO but not SV^91^.

### Recommendation

TEBT should not be used to monitor RBC transfusions. TEBT may be helpful in monitoring longitudinal CO changes during and after RBC transfusions. More research is required prior to routine clinical use.

## TEBT to predict outcome

Few studies are available correlating TEBT with neonatal outcome (supplementary data).

Infants with a birth weight <1250 g who had a low TEBT-CO on day 1 after birth followed by a significant increase on day 2 were shown to have a higher risk of developing IVH and/or NEC^92,93^. A larger increase in CO over the first 48 h after birth has been associated with early brain damage, represented by high grade IVH (IVH ≥ grade 2)^94^. TEBT-CO monitored after PDA ligation was found to not be associated with poorer neurodevelopmental outcome^95^.

TEBT was able to predict IVH in preterm infants at 6 h of age with moderate precision and sensitivity but poor specificity in a deep machine learning study^96^.

### Recommendation

TEBT should not be used to predict neonatal outcomes. More research is required.

## TEBT for monitoring therapeutic hypothermia

Perinatal asphyxia may lead to multi-component hemodynamic compromise – myocardial dysfunction, decreased contractility, reduced preload and afterload, pulmonary hypertension, myocardial ischaemia and decreased CO^97^. These effects may be exacerbated during the hypothermic or re-warming phases of therapeutic hypothermia. EBT may therefore be useful for continuous monitoring.

Few studies are available investigating the use of TEBT during therapeutic hypothermia (supplementary data).

CO was shown to decrease due to a trend towards a decrease in HR with no significant change in SV in the first 6 h of TH initiation^98,99^. CO has been shown to decrease during TH^100^ and increase during rewarming^100,101^. CO has been shown to differ between different grades of HIE in neonates undergoing TH. CO has been shown to be similar in control and mild HIE groups over the first 24 h after birth but differed significantly between mild and moderate HIE groups^102^.

Low SV has been associated with unfavorable outcome^103^ as well as being associated with an abnormal MRI^103^ in infants with HIE. An abnormal MRI in infants with HIE who underwent TH was also associated with lower CO and higher SVR^92^.

### Recommendation

TEBT should not be used to guide clinical management during TH. TEBT may be helpful in monitoring longitudinal CO changes during TH, but more research is required.

## TEBT to monitor cardiac and other surgery

Few studies have utilized TEBT during cardiac surgery (supplementary data).

TEBT-SV, monitored 72 h post-operatively after a TGA switch procedure, showed poor precision as compared to TTE^52^.

TEBT has been stated to be able to monitor CO during prostaglandin therapy and balloon atrial septostomy in hypoplastic left heart syndrome, whilst TTE was unable to provide CO values due to structural abnormalities^104^.

TEBT-CO did not change during application of different intrathoracic insufflation pressures required for thoracic surgery (trachea-esophageal fistula without cardiac defects)^105^.

### Recommendation

TEBT should not be used to monitor neonates undergoing any form of surgery. There is poor and contradictory evidence for its use in this regard.

## TEBT to monitor anesthesia in neonates

General as well as epidural anesthesia may compromise CO in neonates^106,107^. The association between neurological adverse outcomes and neonatal anesthesia remains controversial^108^. It may therefore be beneficial to monitor multiple hemodynamic parameters during neonatal anesthesia and surgery.

TEBT has been used in few neonatal studies in general anesthesia^109–111^ (supplementary data).

TEBT monitoring during induction of anesthesia showed a reduction in CI 1 min or more prior to changes in HR and systolic blood pressure^110^. During general anesthesia in children, including neonates, continuous TEBT monitoring showed that desaturations were associated with decreases in SV index (SVI) with no effect on CI^109^.

Etomidate induction in neonates/infants with congenital heart disease showed no CI changes^111^. CI also did not change pre and post caudal block in infants (including preterm infants) undergoing minor abdominal surgery^112^. The researchers acknowledged that TEBT should be considered as a trend monitor with limitations in measurement of acute hemodynamic changes and low flow states.

### Recommendation

TEBT should not be used to monitor neonates undergoing anesthesia due to contradictory data. Hemodynamic monitoring during neonatal anesthesia and surgery is feasible and may provide insights into longitudinal hemodynamic changes. However, it is limited by the lack of evidence to determine acute changes. This requires more research prior to use in clinical practice.

## TEBT for pharmacological monitoring

Hemodynamic monitoring has been used to quantify drug reactions^112,113^. Due to the possibility of various hemodynamic effects of medications on the neonate, this may improve safety and allow for patient-specific modifications in therapy.

Few and varying pharmacological studies have utilized TEBT (supplementary data).

Caffeine has been shown to decrease many prematurity-related diseases^113^. Neither routine nor early caffeine (<2 h of age) has been shown to affect CO as measured by TTE or TEBT^114^.

Sodium bicarbonate is often used in persistent metabolic acidosis with the assumption that resolution of acidosis may improve cardiac contractility^115^. TEBT-CO showed no improvement in CO during sodium bicarbonate administration despite cerebral and systemic vasodilatation^116^.

Intubation premedication (atropine, morphine/fentanyl, and cisatrucurium) showed no change in TEBT-CO as well as showing no association between changes in BP and CO^56^.

An interventional hemodynamic trial assessing the effectiveness of inhaled nitric oxide or milrinone for pulmonary hypertension was terminated early due to futility, but showed no differences in CO and SVR between treatment and placebo groups^117^.

### Recommendation

TEBT should not be used to monitor hemodynamic parameters during or after any type of pharmacological management.

## TEBT to monitor body position effects on CO

Few studies have utilized TEBT to determine CO changes during body position changes (supplementary data).

TEBT-CO and SV have been shown to decrease with an increase in SVR index (SVRI) in healthy term and preterm LBW infants when turned prone and to return to baseline when turned supine again^118,119^.

In infants with respiratory disorders, prone positioning has been shown to improve CO parameters in infants with RDS, but less so in infants with evolving BPD^120^.

### Recommendation

TEBT may be able to be used for monitoring CO in various body positions but more research is required prior to routine implementation.

## TEBT during neonatal transport

Very few studies have utilized TEBT during transport (supplementary data).

TEBT was shown to be feasible for ICU transports of neonatal and pediatric patient with transport itself not affecting the accuracy of TEBT^121^.

### Recommendation

TEBT should not be used to monitor hemodynamics during transport due to a lack of evidence.

## Factors affecting TEBT measurements

TEBT-derived SV and CO have been shown to be variably affected by maternal and neonatal factors: maternal diabetes^75^, modes of delivery (normal vaginal deliveries vs caesarean section)^66,74^, sex^70^, gestational age groups^42–45^ and birth weight^44,45,63^. Older technologies have also suggested that severe tachycardia may affect accuracy^33^.

Volume expansion, phlebotomies, and hematocrit have been shown to affect TEBT^32,122^. In a study, using older technology, the thoracic segment length was also noted to significantly affect TEBT-CO measurements, with a 3-fold increase in CO depending on which thoracic segment length calculation was used^122^.

Electrode position has been shown to affect measurement parameters in bioimpedance body weight measurements^38,64,123^. It is unknown whether electrode position may affect CO parameters when measuring CO and SV. The accuracy of anthropometric measurements has also been shown to affect how TEBT monitors calculate CO, where for every 1 cm change in length resulted in an SV change of 1.8–36% for preterm infants and 1.6–2% for term infants. Also, for every 100 g in weight change/error, the SV changed 5–7.1% for preterm infants and 1.5–1.8% for term infants for all weight ranges^124^.

Although TEBT has been stated to be feasible in various studies^34,44,121^, signals could not be acquired within the first minute in DCC and resuscitation studies^68,77^ and could take up to 3 min to be acquired^67^. Only 2 studies have looked at signal quality during continuous use of TEBT and showed poor maintenance of high signal quality^68,69^ with improvement only when TEBT data were averaged over 1 minute^68^. Few studies have been performed in neonates to determine signal artefacts but movement artefacts in infants, with subsequent electrode detachment and data loss, have been documented^125^.

Electrode durability may also affect signal quality and therefore reliability of TEBT values, although inter-patient variability also exists^39,45^. TEBT sensor size and placement may pose limitations on placement of other monitoring electrodes^34^. No skin breakdown has been noted with TEBT sensors^73^, but prolonged placement, similar to ECG sensors, may cause skin irritation as the sensors are similar^126^.

Significant differences exist in commercially available technologies based on published algorithms^17,18,20^. Available data for normal and reference ranges for CO and SV differ between technologies^42,63,73^. Older algorithms have also suggested that hematocrit may influence accuracy^127,128^ but has not been assessed in the newer algorithms.

There is also ongoing debate as to the true origin of TEBT signals, theoretical thorax models, and the effects of respiratory and movement artefacts^129^. Device-specific knowledge and continued algorithm adaptation is required and may contribute to future computer-aided diagnoses^130^.

### Recommendation

TEBT may be dependent on various technology-related factors. Anthropometric data should be accurately entered into the monitor and sensor placement should be constantly evaluated. Technology-specific and disease-specific normal/ reference ranges require further research.

## TEBT to monitor thoracic fluid content

EBT monitors are mostly utilized for monitoring CO and SV, together with SVR. However, TFC is another parameter that is present for monitoring by EBT. TFC is the sum of extravascular, intravascular and intrapleural fluid. In neonates during the transition phase, in the absence of a hydrops or other significant edema, it can be assumed that intrapleural fluid is absent. Pulmonary blood flow increases dramatically from intra- to extra-uterine life stabilizing after a few minutes, when functional residual capacity has been established^131^. Therefore, with the intrapleural component being negligible and the intravascular component stabilizing within the first minutes after birth (assuming adequate lung recruitment), TFC equates to extravascular lung fluid in the neonate.

TFC has been used to predict outcomes in critically ill children^132^, monitor fluid responsiveness in children with shock^133^ as well as monitoring fluid overload during hemodyalisis^134^. Few such studies are available in neonates (supplementary data).

TFC reference ranges show increasing TFC values with increasing gestational age^63^. TFC has also been shown to decrease in the first 15 min after delivery^74^, as well as during the subsequent 72–96 h^63,135^.

A few studies have studied TEBT-derived TFC during respiratory distress in neonates^136–138^. TFC may be able to be used as a tool to predict respiratory distress at birth and persisting to 24 h^137^ as well as differentiate RDS and TTN^136,138^. TFC cut-off values have been determined for predicting mechanical ventilation and bronchopulmonary dysplasia^139^.

TFC has also been shown to differ significantly between preterm infants post-RBC transfusion, and non-transfused infants^91,140^. TFC has also been shown to be able to differentiate between infants with hsPDA and those with restrictive or closing PDA^141^.

### Recommendation

TEBT may be helpful in assessing longitudinal TFC changes. However technological differences and lack of normative data require further research prior to routine clinical use.

## WHOLE-BODY EBT

Few studies using WBEBT have been performed in neonates (supplementary data).

Accuracy studies of WBEBT, compared to TTE, in infants showed contradictory evidence of accuracy and precision^142–144^.

Few clinical studies have utilized WBEBT. WEBT-derived SI and CI were shown to increase with increasing PDA size in preterm infants^145^.

### Recommendation

WBEBT should not be used in neonates for CO monitoring due to a lack of evidence of accuracy. Further research comparing WBEBT to a standard reference method should be performed.

## EBT in research

Comparison of CO and SV using the current commercially available EBT devices is not possible, and the results are difficult to pool. Algorithmic differences exist between technologies^146^, equating to a need for technology-specific normative data.

Significant heterogeneity exists in data reporting. Measurement units for CO (ml/kg/min, l/min), SV (ml, ml/kg) and CI (ml/kg/min, ml/min/m^2^) differ. Data reporting should be standardized with consistent units of measurement, preferably indexed to weight, to enable better clinical interpretation and application.

Device accuracy analysis should be performed using Bland Altman not correlation statistics. Trending accuracy should be reported as polar plots not as correlation or changes between time points statistics. This would allow improved comparison of data across studies.

Numerous studies have used TEBT to longitudinally track hemodynamic variables but have used variable time periods, absolute changes, percentage changes as well as changes from baseline^45,52,55–58,63,72,73,80,88,91–93,98,100–102,104^. Future studies could utilize this technology to study longitudinal hemodynamic changes after interventions or for disease progression/improvement but require standardization of change parameters (i.e., changes from baseline) to allow individualization of management as well as improved comparison of study data. Longitudinal hemodynamic changes should be defined a priori. Consideration should be given to what may be clinically applicable changes from baseline (e.g., 20% change in CO from baseline) indicative of improvement or deterioration or response to therapy.

EBT-derived CO and SV can currently only be recommended in the context of research. Absolute value comparison of CO, SV, SVR, and TFC between studies cannot be recommended.

## Discussion

EBT offers a non-invasive, minimal expertise required solution for hemodynamic monitoring of CO, SV, SVR, and TFC. With hemodynamic monitoring, the goal of early identification of systemic decompensation may be achievable, which may in turn, if adequately treated, improve neonatal outcomes. Non-invasive measurement of CO would be ideal in the NICU as it would enable continuous measurement of a parameter that reflects systemic perfusion. Numerous data have shown the inability of clinical and laboratory data to predict or timeously detect hemodynamic compromise in neonates^25^. Ensuring adequate CO, and thus systemic perfusion, may prevent numerous adverse outcomes^6^.

However, there are numerous limitations to the use of current EBT systems for routine use in clinical practice.

EBT, as compared to TTE, lacks accuracy and precision. TTE, commonly used in neonatology^147,148^, is itself inaccurate^149^ and does not represent a true reference standard for accuracy comparison. However, there are currently no better reference methods with which to determine EBT’s accuracy in neonates. Despite numerous studies regarding absolute accuracy of TEBT, only two studies have investigated trending accuracy and showed a poor trending ability. Future research should aim at determining a more accurate comparator for these devices to establish the true accuracy of EBT.

Many studies have reported hemodynamic parameters in various units of measurement. This should be standardized across research to enhance comparison and understanding as to applications in clinical practice (e.g., CO in ml/kg/min and SV in ml). Data analysis also varies with variable reporting methods, despite set standards for data reporting^150,151^. These methodological flaws also need to be addressed in future research.

In TTE, low CO has been defined^62^. However, due to the inaccuracies of EBT and varying technologies, this value may not be relevant. Further research is required to determine what TEBT- or WBEBT-values may represent a low CO requiring intervention. The use of the patient’s baseline measurements to determine individual variations and changes from baseline may be of more use, clinically, than absolute values. This requires more research.

The wide application of EBT, as well as ongoing research of use of TEBT in monitoring RBC transfusion for anemia^45,152,153^, respiratory disease monitoring^154^, ventilation monitoring^155,156^ and determining end-organ perfusion^157^, is a testament to the need for EBT-determined CO monitoring in neonates. However, the heterogenicity of studies consisting of infants of different gestation, postnatal age, birth weights, undergoing various physiological changes and pathophysiological processes and subjected to various interventions, contributes to the inability to determine normality and determine the effect of abnormality in these studies^49,51^. It also complicates the ability to accurately compare the accuracy of these devices. Although this would be representative of the “true state” of the neonates who most require CO monitoring, it complicates the ability of using these devices in routine practice. Research in the use of WBEBT in neonates is also ongoing, showing the continuing need for improved EBT technology and continuing need for non-invasive CO monitoring^158^.

Similar to problems encountered in NIRS monitoring^159^, this lack in accuracy may suggest that each patient (neonate) should be used as their own control, and changes from an individual baseline may provide information that could be used in clinical practice. Few neonatal studies have used changes from baseline to determine individual patient response to management or medical events^80^. One such paediatric study does exist^106^, which showed large ranges in intra-individual variability (46–56%) with changes from baseline varying between 62% and 126%. These changes may represent biological variability requiring an individualized approach to CO monitoring. This needs to be further researched with the inclusion of long-term outcomes to determine effects of physiology.

The heterogeneity of patient populations, diverse disease processes, and intra-individual variability may lend itself to the practice of precision medicine in neonatology, enabling precise and patient-specific tailored management of a disease^160^. TEBT may enable this approach, allowing for individualized hemodynamic management^161^. More research, however, is still required to overcome the current lack of accuracy and trending ability, lack of normal or reference ranges, and lack of data of what constitutes a clinically relevant change in baseline. TTE should be performed in all neonates where EBT is used, to determine cardiac anatomy, and function as well as to verify any changes in EBT.

Few neonatal studies are available showing that specific hemodynamic monitoring leads to improved outcome. This is also true of adult medicine^162^. Similar to adults, there is a need for disease-specific and process-specific research and clinical trials to use patient-centered outcomes to effectively monitor patients and rapidly detect instability. Some adult goal-directed fluid management continuous CO monitoring studies have shown decreased morbidities^163^, decreased cost^164,165^, no increase in costs^163^ and decrease in hospitalization and hospitalization-associated costs^166^. A systematic review showed that pre-operative hemodynamic optimization via various methods, was cost effective, would increase efficiency of health systems, and decrease the burden on public health systems^156^. No such studies have been performed in children or neonates. There may, therefore, be an economic advantage in using TEBT. Further research is required in this field.

## Conclusion

EBT holds potential for CO monitoring and improvement in neonatal hemodynamic care. However, a lack of accuracy, lack of safety parameters (what EBT-CO is too low?), biological variability and diversity of reported research data negate the possibility for the recommendation of the use of EBT in routine clinical practice.

Further technical improvement and deeper understanding of technological algorithms are required. Further research is required before EBT can be recommended to be implemented into routine clinical practice.

EBT should currently only be used in research to determine accuracy, safety margins and long-term clinical outcomes based on these parameters.

Numerous research opportunities exist in EBT-determined hemodynamic monitoring (Table 4) and require attention prior to rolling out these monitors to routine practice.

**Table 4:** Research requirements in EBT.

Disease/Process	Barrier	Research requirements
Accuracy	Lack of accurate comparator	Determine a reference comparator to determine true accuracy of EBT
	Data methodological issues	Standard reporting of accuracy and precision (Bland Altman, bias, LOA) and trending data (polar plots – concordance, angular bias, angular concordance)
	Inconsistencies in parameters measured (CO, SV) and variable units of measurement	Units of measurement should be standardized across research (indexed to weight – ml/kg/min)
CO monitoring in cardiac physiology/abnormalities	Uncertainty regarding the effect of physiological changes	Develop methods in which to determine baseline values and variations in baseline values in the presence of physiological shunts (PDA, PFO)
	Pathophysiological changes	Develop methods to determine the effects of various congenital and acquired cardiac anomalies on EBT (CHD, PPHN, myocardial dysfunction, etc.)
	Interventional practices	Develop methods to determine changes from baseline pre-and post-treatment for cardiac disease (PDA treatment, cardiac surgery)
CO monitoring in respiratory anomalies	Lack of standardization of research design to determine effects of positive pressure ventilation on neonates	Consistent reporting of ventilation (pressure) parameters
Hemodynamic monitoring	Lack of standardization of research design, data analysis and long-term outcome determination	Increased research in disease -specific and population-specific populations with long-term follow-up
Outcome prediction	Uncertainty regarding clinical variables to determine a clinically significant deviation from baseline	Determine a clinically applicable deviation from baseline parameters

*CHD* congenital heart disease, *CO* cardiac output, *EBT* electrical biosensing technology, *LOA* limits of agreement, *PDA* patent ductus arteriosus, *PFO* patent foramen ovale, *PPHN* persistent pulmonary hypertension, *SV* stroke volume.

## Supplementary information

Supplementary data
