# Correction: A recommendation for the use of electrical biosensing technology in neonatology

**DOI:** 10.1038/s41390-024-03446-3

**Published:** 2024-08-29

**Authors:** Lizelle van Wyk, Topun Austin, Bernard Barzilay, Maria Carmen Bravo, Morten Breindahl, Christoph Czernik, Eugene Dempsey, Willem-Pieter de Boode, Willem de Vries, Beate Horsberg Eriksen, Jean-Claude Fauchére, Elisabeth M. W. Kooi, Philip T. Levy, Patrick J. McNamara, Subhabrata Mitra, Eirik Nestaas, Heike Rabe, Yacov Rabi, Sheryle R. Rogerson, Marilena Savoia, Frederico Schena, Arvind Sehgal, Christoph E. Schwarz, Ulrich Thome, David van Laere, Gabriela C. Zaharie, Samir Gupta, ESPR Special Interest Group on Non-Invasive Cardiac Output Monitoring, Topun Austin, Bernard Barzilay, Maria Carmen Bravo, Morten Breindahl, Christoph Czernik, Eugene Dempsey, Beate Horsberg Eriksen, Jean-Claude Fauchére, Elisabeth M. W. Kooi, Philip T. Levy, Patrick J. McNamara, Subhabrata Mitra, Eirik Nestaas, Heike Rabe, Yacov Rabi, Sheryle R. Rogerson, Marilena Savoia, Frederico Schena, Arvind Sehgal, Christoph E. Schwarz, Ulrich Thome, Gabriela C. Zaharie, Samir Gupta, Lizelle van Wyk, Willem-Pieter de Boode, Willem de Vries, David van Laere

**Affiliations:** 1 Department of Paediatrics and Child Health, Faculty of Medicine and Health Sciences, Stellenbosch University and Tygerberg Hospital, Cape Town, South Africa; 2 Neonatal Intensive Care Unit, Rosie Hospital, Cambridge University Hospitals NHS Foundation Trust, Cambridge Biomedical Campus, Cambridge, UK; 3 Neonatal Intensive Care Unit, Assaf Harofeh Medical Center, Tzrifin, Israel; 4 Department of Neonatology, La Paz University Hospital and IdiPaz, Madrid, Spain; 5 Department of Neonatology, Copenhagen University Hospital, Rigshospitalet, Copenhagen Denmark; 6 Department of Neonatology, Charité - Universitätsmedizin Berlin, Berlin, Germany; 7 Department of Paediatrics and Child Health, University College Cork, Cork, Ireland; 8 Department of Neonatology, Radboud University Medical Center, Radboud Institute for Health Sciences, Amalia Children’s Hospital, Nijmegen, The Netherlands; 9 Division of Woman and Baby, Department of Neonatology, University Medical Centre Utrecht, Wilhelmina Children’s Hospital, Utrecht University, Utrecht, The Netherlands; 10 Department of Paediatrics, Møre and Romsdal Hospital Trust, Ålesund, Norway; 11 Clinical Research Unit, Norwegian University of Science and Technology, Trondheim, Norway; 12 Department of Neonatology, University Hospital Zurich, University of Zurich, Zurich, Switzerland; 13 Division of Neonatology, Department of Pediatrics, Beatrix Children’s Hospital, University of Groningen, University Medical Centre, Groningen, The Netherlands; 14 Department of Newborn Medicine, Boston Children’s Hospital, Boston, MA USA; 15 Department of Pediatrics, Harvard Medical School, Boston, MA USA; 16 Department of Paediatrics, University of Iowa, Iowa City, IA USA; 17 Institute for Women’s Health, University College London, London, UK; 18 Institute of Clinical Medicine, Faculty of Medicine, University of Oslo, Oslo, Norway; 19 Clinic of Paediatrics and Adolescence, Akershus University Hospital, Lørenskog, Norway; 20 Brighton and Sussex Medical School, University of Sussex, Brighton, UK; 21 University of Calgary, Alberta, Canada; 22 Newborn Research Centre, The Royal Women’s Hospital, Melbourne, VIC Australia; 23 Department of Obstetrics and Gynaecology, The University of Melbourne, Melbourne, VIC Australia; 24 Neonatal Intensive Care Unit, S Maria Della Misericordia Hospital, Udine, Italy; 25 Ospedale Maggiore Policlinico, Milano, Italy; 26 Monash Newborn, Monash Children’s Hospital, Melbourne, VIC Australia; 27 Department of Paediatrics, Monash University, Melbourne, VIC Australia; 28 Department of Neonatology, Center for Pediatric and Adolescent Medicine, University of Heidelberg, Heidelberg, Germany; 29 Division of Neonatology, Department of Pediatrics, University of Leipzig Medical Centre, Leipzig, Germany; 30 Neonatal Intensive Care Unit, Universitair Ziekenhuis, Antwerp, Belgium; 31 Neonatology Department, University of Medicine and Pharmacy, Iuliu Hatieganu, Cluj -Napoca, Romania; 32 Department of Engineering, Durham University, Durham, UK; 33 Division of Neonatology, Department of Pediatrics, Sidra Medicine, Doha, Qatar

Correction to: *Pediatric Research* 10.1038/s41390-024-03369-z, published online 08 July 2024

In the original version of the article, the author’s name Christoph E. Schwarz was incorrectly given as ‘Christop E. Schwarz’. The author’s name Arvind Sehgal was incorrectly given as ‘Arvind Seghal’. The original article has been corrected.

